# Supplementary material for: The discovery of novel noncoding RNAs in 50 bacterial genomes
Source: Nucleic Acids Res. 2024 Apr 22;52(9):5152–65. doi: 10.1093/nar/gkae248 (PMC11109978; doi:10.1093/nar/gkae248)
Supplement: gkae248_Supplemental_Files [file gkae248_supplemental_files.zip › Narunsky 50 Genomes RNA Motifs Files.zip!/Publication/56-D_turgidum/Figures/LRC-56-38-NC_011661_1_IGR_113_1386834_1387003.sto.pdf]

LRC-56-38-NC_011661_1_IGR_113_1386834_1387003.sto.r2r

5′-○○UUUUCACCUCCUUYYY○●UU●●●UYAAA●AUAAGGGAAAGCCUYCUGGCUUUCCCUUA●●●A●○○○○○○○●U●Y●UA●●●UUYGA○ARACCAG●AAGGCCUACGUAGGAAAU●○U●●●●ACCUACGGUCUUCGGCCC○○○●●AU●●YAR●●UUUUAYUUU○○
